# *Bordetella pertussis* in children hospitalized with a respiratory infection: clinical characteristics and pathogen detection in household contacts

**DOI:** 10.1186/s13104-018-3405-7

**Published:** 2018-05-18

**Authors:** Juana del Valle-Mendoza, Wilmer Silva-Caso, Miguel Angel Aguilar-Luis, Cristina del Valle-Vargas, Erico Cieza-Mora, Johanna Martins-Luna, Ronald Aquino-Ortega, Andrea Silva-Vásquez, Jorge Bazán-Mayra, Pablo Weilg

**Affiliations:** 1 Research and Innovation Centre of the Faculty of Health Sciences, Universidad Peruana de Ciencias Aplicadas, Lima, Peru; 2 Laboratorio de Biología Molecular, Instituto de Investigación Nutricional, Lima, Peru; 3 Instituto de Investigación de Enfermedades Infecciosas, Lima, Peru; 4 Facultad de Medicina, Univesidad de Barcelona, Barcelona, Spain; 5 Servicio de Pediatría, Hospital Docente Regional de Salud de Cajamarca, Cajamarca, Peru; 6 Laboratorio de Referencia, Dirección Regional de Salud de Cajamarca (DIRESA), Cajamarca, Peru

**Keywords:** Pertussis, *Bordetella pertussis*, Whooping cough, PCR, Peru

## Abstract

**Objective:**

Describe the prevalence of *Bordetella pertussis* via PCR in children under 5 years old hospitalized as probable cases of pertussis and report the most common clinical features among them.

**Results:**

A positive PCR result for *B. pertussis* was observed in 20.5% of our samples (18/88), one-third of them were from infants between 2 and 3 months old. The most common symptoms were paroxysms of coughing (88.9%), difficulty breathing (72.2%), cyanosis (77.8%) and fever (50%). The mother was the most common symptomatic carrier (27.8%), followed by uncles/aunts (22.2%) among children with pertussis.

**Electronic supplementary material:**

The online version of this article (10.1186/s13104-018-3405-7) contains supplementary material, which is available to authorized users.

## Introduction

Pertussis, also known as ‘whooping cough’, is an acute respiratory tract infection caused by the gram-negative bacteria *Bordetella pertussis* [1–3]. Worldwide, around 30 million cases of pertussis and 160,000 deaths in children younger than 5 years old are registered every year, 90% of them occurring in developing countries [4–6].

In the last years, despite a widespread vaccination, the resurgence of *B. pertussis* infections has been observed, primarily affecting low-income countries [7–11]. Despite the pertussis vaccine success, cyclical outbreaks are observed every 2–5 years as they did in the prevaccine era [12, 13]. Furthermore, the vaccination calendar has left a window of vulnerability for newborns and infants in which high morbidity and mortality rates are observed [14–17]. In addition, in infants younger than 3 months and neonates the increasing incidence of this infection and the sustained mortality rates have encouraged further investigation about the efficacy and safety of pertussis vaccination for pregnant women as an attempt to reduce the disease burden [18].

In Peru, since 2017 a rapid increase of *B. pertussis* has been observed and by the first half of the year, the number of cases has doubled compared to the previous year suggesting the possibility of a new outbreak [19]. This study main objective was to describe the prevalence of *B. pertussis* via PCR in children under 5 years old hospitalized as probable cases of pertussis and report the most common clinical features among them.

## Main text

### Materials and methods

#### Patients

A cross-sectional study was conducted in Cajamarca in coordination with the “*Dirección Regional de Salud de Cajamarca, Peru*”. Cajamarca region is located in the Andes Mountain Range and was the second most affected region by *B. pertussis* in 2016 [19].

Children under 5 years old hospitalized as probable cases of pertussis at the *Hospital Regional de Cajamarca* were consecutively studied from April 2016 to September 2017. Household contacts with similar respiratory complaints were also included in the study and were considered positive after PCR detection of *B. pertussis*.

Cases were defined as probable for pertussis in the absence of a more likely diagnosis of cough illness with one of the following symptoms: Paroxysms of coughing or inspiratory “whoop” or posttussive vomiting or apnea as per CDC case definition recommendations [20].

This study was approved by the Research Ethics Board of the *Hospital Docente Regional de Cajamarca*, Peru. A written informed consent was signed by parents or children caregivers before enrollment. Household contacts also signed a written informed consent before enrollment.

#### Samples

Nasopharyngeal samples were obtained inserted one swab into each nostril parallel to the palate (calcium alginate swab, USA) and submerged into transport solution (phosphate buffered saline).

#### DNA extraction

DNA was extracted from a volume of 200 μl of each samples using a commercial kit (High Pure Template Preparation Kit, Roche Applied Science, Germany) according to the manufacturer's instructions.

#### Real-time PCR assay detection *Bordetella pertussis* with the TaqMan probe

PCR was performed using a BHQ quencher probe at 125 and 250 nM of primers in a final volume of 20 μl. Five microliters of the extracted DNA were combined with 15 µl of the master mix. PCR conditions for *B. pertussis* were 95 °C for 10 s, 60 cycles of 5 s at 95 °C, 5 s at 57 °C and 30 s at 72 °C. All cycles were performed in Light Cycler^®^ 2.0 (Roche Diagnostic, Deutschland-Mannheim, Germany). The primers and the probe used were described by Kosters et al. [21].

#### Statistical analysis

Quantitative variables were described as frequencies and percentages for each group using the GraphPad Prism3 statistical (Graph Pad Sofware Inc., San Diego, USA).

### Results

A total of 88 children under 5 years old hospitalized as probable cases of pertussis were prospectively studied from April 2016 to September 2017. In our study population, more than 70% of patients were infants under 3 months old, with infants between 29 days to 2 months old being the most predominant age group in 31.8% of patients. No significant difference between gender was observed among our patients as 53.4% were male vs 46.6% females (Table 1).

**Table 1:** Demographics of patients with whooping cough syndrome and *Bordetella pertussis*

Characteristics	Total of patients n = 88 (%)	Patients positive for *B. pertussis* n = 18 (%)
Age		
< 28 days	13 (14.8)	2 (11.1)
29 days–< 2 months	28 (31.8)	3 (16.7)
2–< 3 months	21 (23.9)	6 (33.3)
3–5 months	10 (11.4)	2 (11.1)
6–11 months	6 (6.8)	2 (11.1)
1–5 years	6 (6.8)	3 (16.7)
Gender		
Male	47 (53.4)	13 (72.2)
Female	41 (46.6)	5 (27.8)
Household contacts		
Mother	14 (15.9)	5 (27.8)
Father	7 (8)	3 (16.7)
Siblings < 7 years old	14 (15.9)	3 (16.7)
Siblings 7–10 years old	7 (8)	2 (11.1)
Siblings > 10 years old	2 (2.3)	1 (5.6)
Uncles/aunts	6 (6.8)	4 (22.2)
Others	11 (12.5)	3 (16.7)

A positive PCR result for *B. pertussis* was observed in 20.5% of our samples (18/88), one-third of them were from infants between 2 and 3 months old followed by three positive cases on infants between 29 to 2 months old and three cases in children between 1 and 5 years old. Most of our patients with a positive sample were male infants in 72.2% of cases **(**Table 1).

Our patients were hospitalized as probable cases of ‘whooping cough’ and the most common presenting symptoms were paroxysms of coughing (76.1%), difficulty breathing (75%), cyanosis (67%) and fever (52.3%). The same symptoms were also observed among patients with positive samples for *B. pertussis* in which paroxysms of coughing (88.9%), difficulty breathing (72.2%), cyanosis (77.8%) and fever (50%) were the most frequent complaints. Furthermore, clinical symptoms were compared by age groups showing that paroxysmal coughing was the most common symptom across all ages, except in neonates were cyanosis was the most common presentation. Complications during hospitalization were also registered, pneumonia was the most frequent outcome in 33% of our study population and 38.9% of our patients with *B. pertussis* (Table 2). In addition, 83% (15/18) of our patients with *B. pertussis* received antibiotic on day 1 of hospitalization, the same day we enrolled them but before we sampled and reported our results.

**Table 2:** Clinical symptoms among patients with whooping cough syndrome and *Bordetella pertussis*

Clinical symptoms	Total of patients n = 88 (%)	Patients positive for *B. pertussis* n = 18 (%)				
		< 28 days n = 2	29 days–< 3 months n = 9	3–5 months n = 2	6–11 months n = 2	1–5 years n = 3
Paroxysmal cough	67 (76.1)	1 (5.6)	7 (38.9)	2 (11.1)	2 (11.1)	3 (16.7)
Difficulty breathing	66 (75)	1 (5.6)	6 (33.3)	1 (5.6)	2 (11.1)	3 (16.7)
Cyanosis	59 (67)	2 (11.1)	7 (38.9)	1 (5.6)	2 (11.1)	2 (11.1)
Fever	46 (52.3)	1 (5.6)	5 (27.8)	0 (0)	1 (5.6)	2 (11.1)
Posttussive emesis	34 (38.6)	1 (5.6)	4 (22.2)	0 (0)	1 (5.6)	2 (11.1)
Breastfeeding difficulties	32 (36.4)	1 (5.6)	3 (16.7)	1 (5.6)	0 (0)	0 (0)
Ruddiness	21 (23.9)	1 (5.6)	1 (5.6)	1 (5.6)	1 (5.6)	1 (5.6)
Stridor	16 (18.2)	0 (0)	1 (5.6)	1 (5.6)	2 (11.1)	1 (5.6)
Diarrhea	15 (17)	0 (0)	3 (16.7)	0 (0)	0 (0)	1 (5.6)
Apnea	9 (10.2)	0 (0)	2 (11.1)	0 (0)	0 (0)	0 (0)
Complications						
Pneumonia	29 (33)	1 (5.6)	5 (27.8)	0 (0)	0 (0)	1 (5.6)
Acute bronquial obstructive syndrome	18 (20.5)	0 (0)	0 (0)	0 (0)	1 (5.6)	1 (5.6)
Atelectasis	10 (11.4)	0 (0)	2 (11.1)	0 (0)	1 (5.6)	1 (5.6)
Convulsions	4 (4.5)	0 (0)	2 (11.1)	0 (0)	0 (0)	0 (0)
Umbilical hernia	1 (1.1)	1 (5.6)	0 (0)	0 (0)	0 (0)	0 (0)

High leukocyte and lymphocyte count were assessed in our patients. We observed that both leukocytosis and lymphocytosis were present in four cases with *B. pertussis*. On the other hand, leukocytosis and lymphocytosis were also observed in three and four patients with negative results for *B. pertussis* respectively (Additional file 1: Table S1).

From our patients with a positive result for *B. pertussis*, most cases older than 2 months old didn’t receive any vaccination 76.9% (10/13), one patient had the incomplete vaccination and only one patient with a positive sample for *B. pertussis* received the two vaccine doses (Additional file 1: Table S2).

Household contacts who presented with respiratory symptoms were also evaluated for the presence of *B. pertussis*. We found that mothers and siblings under 7 years old were the most common family members with positive samples for *B. pertussis* in our population. This predominance was more evident among patients with *B. pertussis* in which 27.8% (5/18) of their mother were also positive for the bacteria (Table 1).

**Fig. 1:**
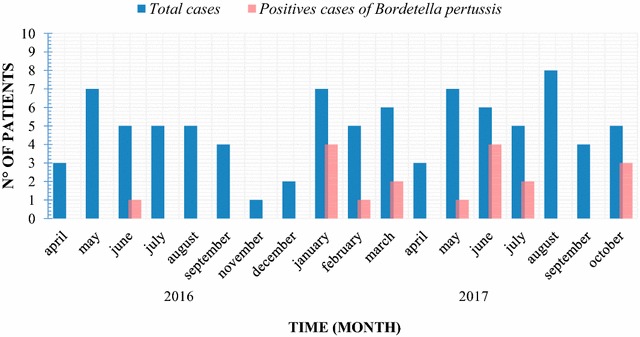
Seasonal distribution among patients with whooping cough syndrome and *B. pertussis*

We enrolled patients with a clinical presentation compatible with whooping cough through the whole study period. However, most of our patients with positive samples for *B. pertussis* were observed during 2017, being January and June the months with the higher case-rate distribution (Fig. 1).

### Discussion

Latin America has experienced a resurgence of *B. pertussis* infections with high morbidity and mortality rates among infants younger than 6 months old, who represent up to 75% of pertussis cases [5, 22–28].

In our study, a total of 18 cases (20.5%) had positive samples for *B. pertussis*, being 70% of them infants under 3 months old. This proportion of positive samples is slightly lower than a previous study we conducted in 2015, where 38.4% of probable cases for pertussis were also positive for the bacteria [27]. However, these variations are expected as the clinical features have shown to be insufficient to establish a diagnosis and it is estimated that without PCR testing, the overall percentage of missed cases would range from 9 to 26% per year in infants under 6-month-old [29].

Even though pertussis clinical presentation tends to be unspecific, there are some clinical features that seem to be more common in different age groups [14, 30]. In our population, we observed that difficulty breathing and cyanosis were present in most of our patients between 29 days to 3 months old; however, we were surprised that paroxysmal coughing was also a common symptom in this age group. Additionally, it also has been reported that post-tussive vomiting is common at all ages [14]; in our series, it was present in 44.4% (8/18) of patients with *B. pertussis*.

It has been demonstrated that a whooping cough alone is not enough to start antibiotics immediately, especially in infants younger than 4 months [5]. However, in rural areas where laboratory resources are limited physician usually give macrolides when there is high suspicious of pertussis. In our population, 17% (15/88) of patients received antibiotics on day 1 of hospitalization before we took samples. In addition, 83% (15/18) of patients with *B. pertussis* were covered before we report their results as positive, and the other three cases were started on antibiotics the same day we sent our results.

One study in Mexican infant showed that 59% of patients with pertussis had leukocytosis and 64.4% presented lymphocytosis, with a mean age of 87.1 days old [31]. In our study, we found that leukocytosis and lymphocytosis were each observed only in 4 (22.2%) pertussis cases. However, these findings were not exclusive among patients with *B. pertussis*, as we found three patients with leukocytosis and four with lymphocytosis in the group of patients with negative samples.

*Bordetella pertussis* is a highly contagious disease acquired through direct contact or inhalation and the source of infection is usually the mother has been identified as the most common source in up to 63% of cases, followed by fathers, siblings and other family members [11, 17, 31]. In our study, among children with *B. pertussis*, we found that mothers were the most common symptomatic carriers in 27.8% (5/18) of patients, followed by uncles or aunts in six cases. Notably, we also found nine mothers with *B. pertussis* among infants with negative samples of the bacteria demonstrating the presence of carriers even in patients without the disease.

Evidence suggests that the households contacts transmission occurs due to the limited immunity to *B. pertussis* infection whether acquired naturally or by immunization [32]. In adult carriers, who usually do not receive booster protection, the levels of protective antibodies almost negligible with more than 50% of them lost in the first 6 years after vaccination [33]. On the other hand, contacts between 0 and 18 years old, primarily represented by the patients’ siblings, are usually carriers without vaccination or incomplete schedules who did not receive chemoprophylaxis [34].

The most recognized strategy is the maternal pertussis immunization supported by the World Health Organization (WHO) which has been found to be highly effective at preventing severe disease in infants [34–37]. However, the effectiveness of pertussis immunization in preventing transmission in other household contacts is mostly unknown [33, 34]. Nevertheless, it is recommended that all contacts who have an incomplete immunization should be vaccinated in addition to receiving chemoprophylaxis. In addition, adolescents and adults, especially those in close contact with children, may benefit from a booster dose of the acellular vaccine. Although, this immunity will wane considerably fast having no impact in a distant future [34].

We analyzed pertussis vaccination status in our population. Not surprisingly, we observed that 76.9% (10/13) our patients older than 2 months old with *B. pertussis* didn’t receive any vaccination. Thus, indicating that children with incomplete vaccination are prone to be infected by the bacteria as we found in two cases. Finally, infants too young to receive the pertussis vaccine represented 27.8% (5/18) of our patients with *B. pertussis*, demonstrating once again the vulnerability window of our immunization calendar.

### Conclusions

*Bordetella pertussis* is an important cause of respiratory tract infection in children under 5 years of age, primarily affecting infants under 6 months old in whom the diagnosis is not always suspected. The vaccination schedule leaves a window of vulnerability for *B. pertussis* infection among infants under 2 months old. In rural areas of Peru, limited laboratory resources may contribute to the underdiagnosis of the disease and an increased use of macrolides upon clinical criteria. In our study, mothers were the most common symptomatic carrier in patients with and without pertussis.

## Limitations

The present study had one important limitations. We designed the study for the detection of *B. pertussis* we cannot exclude the presence of other etiologies that may be responsible for the patients’ clinical presentation.

## Additional file

**Additional file 1: Table S1.** Leukocytosis and lymphocytosis in children with whooping cough syndrome. **Table S2.** The following normal values were used as a reference to determine high counts of white blood cells and lymphocytes in each age group.

## Abbreviations

PCR: polymerase chain reaction
DNA: deoxyribonucleic acid
bp: base pairs
*B. pertussis*: *Bordetella pertussis*
