# Supplementary material for: Bordetella pertussis in children hospitalized with a respiratory infection: clinical characteristics and pathogen detection in household contacts
Source: BMC Res Notes. 2018 May 18;11:318. doi: 10.1186/s13104-018-3405-7 (PMC5960213; doi:10.1186/s13104-018-3405-7)
Supplement: Supplementary file 1 — Additional file 1: Table S1. Leukocytosis and lymphocytosis in children with whooping cough syndrome. Table S2. The following normal values were used as a reference to determine high counts of white blood cells and lymphocytes in each age group. [file 13104_2018_3405_MOESM1_ESM.docx]

**Table S1.** Use of antibiotics and vaccination coverage status among children with PCR confirmed *Bordetella pertussis.*

|  | **Neonates** | **29 days –**  **< 2 months** | **2 - < 4 months** | **4 m - < 12 months** | **1 - 5**  **years** |
| --- | --- | --- | --- | --- | --- |
| **Use of Antibiotics** | n=2 | n=3 | n=6 | n=4 | n=3 |
| Yes | 2 | 2 | 6 | 2 | 3 |
| No | 0 | 1 | 0 | 2 | 0 |
| **Vaccination coverage** |  | | | | |
| 1 dose | - | - | 0 | 1 | 1 |
| 2 doses | - | - | 0 | 1 | 0 |
| 3 doses | - | - | - | 0 | 0 |
| None | 2 | 3 | 6 | 2 | 2 |

**Table S2.** Leukocytosis and lymphocytosis in children with whooping cough syndrome

| ***B. pertussis*** | **Blood counts** | **≤ 28 days** | **29 days –**  **< 3months** | **3 - 5 months** | **6 – 11 months** | **1 - 5 years** |
| --- | --- | --- | --- | --- | --- | --- |
| **Negative n=70 (%)** | **Leukocytosis** | 0 (0.0) | 1 (1.1) | 1 (1.4) | 0 (0.0) | 1 (1.4) |
|  | **Lymphocytosis** | 1 (1.4) | 2 (2.8) | 1 (1.4) | 0 (0.0) | 0 (0.0) |
| **Positive n=18(%)** | **Leukocytosis** | 0 (0.0) | 1 (5.6) | 2 (11.1) | 0 (0.0) | 1 (5.6) |
|  | **Lymphocytosis** | 0 (0.0) | 1 (5.6) | 0 (0.0) | 1 (5.6) | 2 (11.1) |
